# If abortion substantially risks homicide, it should be illegal

**DOI:** 10.1007/s11017-025-09737-y

**Published:** 2025-12-10

**Authors:** Matthew Braddock

**Affiliations:** The University of Tennessee at Martin, Martin, USA

**Keywords:** Abortion, Abortion law, Harm prevention, Harm principle, Moral risk

## Abstract

Is abortion homicide, the morally unjust killing of a person? Should it be illegal? There is widespread disagreement. However, there is a way through the impasse. It is not necessary to establish whether abortion *is* homicide, only whether it *substantially risks* homicide. If abortion presents this risk of harm, then lawmakers have a powerful reason to criminalize it. This paper defends *The Prevention Argument*: if abortion substantially risks committing homicide after 10 weeks’ gestation, then lawmakers should criminalize such abortions to prevent the foreseeable harm of mass homicide. Why 10 weeks? Because it is plausible that abortions after 10 weeks endanger an innocent person’s life and, if so, the harm preventive case for criminalizing them is strong. This argument deserves a hearing because it has a modest burden of proof and relies on accepted normative principles like the harm principle. It could also be extended to earlier abortions.

## Introduction: motivating cases

Consider the following cases:

***Demolition:*** A demolition expert is called in to detonate a building where street children sometimes play. Right before the deadline to blow it up, she detects movement in the building—someone or something is inside. It is probably a stray animal, but it could be one of the street children who somehow got into the building after it was cleared. If she clears the building again, however, she will not meet her deadline. And if she does not meet her deadline, she will lose her job. Now she faces a choice: should she substantially risk killing a child to avoid losing her job?

***Driver***: A woman is running late for an important flight. If she does not make this flight, she will lose her job. Losing her job would interfere with her life and burden her financially, mentally, and physically. In a rush to get to the airport, she speeds through heavy traffic and approaches a long red light at a blind intersection—her view of oncoming cars is completely obstructed. She faces a choice: should she speed through the red light, not knowing whether a car is coming the other way?

***Euthanasia***: A woman is considering whether to euthanize her burdensome child, who was born severely disabled. It is fairly uncertain^1^ whether doing so would be morally permissible or the unjust killing of an innocent person. The woman faces a choice: should she substantially risk wrongfully euthanizing her child to avoid the burdens of childcare or adoption placement?

Such conduct is wrong and should be illegal. Why? Because the agent substantially risks harm to others, namely *homicide*—the morally unjust killing of a person with a right to life. It is wrong and should be illegal to take such a risk, unless one has overriding justification. The agents in these cases, however, have no such justification.

Notice that the cost to the agent does not justify their endangering conduct. The expected costs of losing one’s job or caring for a disabled child—the financial insecurity, mental burdens, physical hardships, and loss of autonomy—do not justify endangering another person’s life.

Notice that the actual outcome of their endangering conduct cannot justify it. It should be illegal for the demolition expert to blow up the building even if, when the dust settles, she discovers that she killed a dog (not a child), even if nobody can establish who or what she has killed. It should be illegal for the driver to speed through the red light, even if she gets lucky and does not kill anyone. In fact, such conduct is illegal across jurisdictions. Even if no car was in fact coming the other way, the driver would still be guilty of *reckless endangerment*—reckless conduct that substantially and unjustifiably risks serious harm to other persons. If the outcome is a dead child, the demolition expert would be guilty of criminal homicide (i.e. manslaughter). But if the outcome is a dead dog or if nobody can establish who or what she has killed, she would still be guilty of reckless endangerment. Both homicide and endangerment are serious crimes.

Such cases motivate a normative legal argument against abortion—*The Endangerment Argument:*

Premise: Abortion substantially and wrongfully risks committing homicide.

Premise: If abortion substantially and wrongfully risks committing homicide, then it should be illegal.

Conclusion: Therefore, abortion should be illegal.

In this paper, I motivate the first premise and develop a sustained argument—*The Prevention Argument*—for the second premise.

Is abortion homicide, the morally unjust killing of a person? Should it be illegal? There is widespread disagreement. However, there is a way through the impasse. It is not necessary to establish whether abortion *is* homicide, only whether it *substantially risks* homicide. If abortion presents this risk of harm, then lawmakers have a powerful reason to criminalize it. This paper defends *The Prevention Argument*: if abortion substantially risks committing homicide after 10 weeks’ gestation, then lawmakers should criminalize such abortions to prevent the foreseeable harm of mass homicide. Why 10 weeks? Because it is plausible that abortions after 10 weeks endanger an innocent person’s life and, if so, the harm preventive case for criminalizing them is strong. This argument deserves a hearing because it has a modest burden of proof and relies on accepted normative principles like the harm principle. It could also be extended to earlier abortions.

I should briefly situate this paper in the abortion debate. Most agree that there are moral and legal limits on personal liberty. One cannot, for example, kill an innocent person in the name of one’s right to bodily autonomy; “Your right to swing your fist ends where another person’s nose begins,” the old saying goes. Accordingly, the abortion debate has focused on whether abortion is the killing of an innocent person with a right to life. To justify abortion rights, courts and legislatures have consistently relied, at least implicitly, on the claim that preborn human beings are not persons.^2^

However, there is growing discussion of a different question: what if abortion *substantially risks* the unjust killing of a person, or *endangers* a person’s life? According to moral risk arguments, abortion is wrong because it risks committing a serious wrong like homicide [3–10]. Are there normative legal implications to this? Most agree that one cannot substantially risk homicide in the name of one's right to bodily autonomy, liberty, property, and so on. One cannot, for example, drive drunk, shoot unidentified targets, demolish buildings without checking whether a person is inside, etc. If abortion substantially risks homicide, should it be illegal too? So far, there has been little discussion of the legal implications [2, 4, 6–9]. This paper will trace them.

In this section, I introduced motivating cases. Next, I explain the main argument—*The Prevention Argument*. In the rest of the paper, I support the premises. Along the way I address expected objections: the good Samaritan argument, the self-defense justification of abortion, the objection that making abortions illegal won’t stop them, and the backstreet abortion objection.

## The prevention argument

This paper’s main argument is *The Prevention Argument*:

1. Abortion substantially and wrongfully risks committing homicide after 10 weeks’ gestation (exception: mother’s life cases).
2. Homicide is a serious harm.
3. *Harm Principle*: Lawmakers have good reason to criminalize conduct that wrongfully causes harm (or substantially and wrongfully risks causing harm) to other persons, if criminalization would prevent foreseeable harm and the harm prevented outweighs the harm created.
4. Criminalizing abortions after 10 weeks’ gestation would effectively prevent them.
5. Therefore, criminalization would prevent foreseeable harm, namely mass homicide. [From 1, 2, and 4]
6. The foreseeable harm prevented by criminalization outweighs the harm created, especially if criminalization is combined with social policies that effectively support women.
7. Thus, lawmakers have good reason to criminalize abortion after 10 weeks’ gestation and to combine criminalization with social policies that effectively support women. [From 1–6]
8. There is no overriding reason to refrain.
9. Thus, lawmakers should criminalize abortion after 10 weeks’ gestation and combine criminalization with social policies that effectively support women. [From 7 and 8].

For clarification, it is worth defining terms:

*Abortion* refers to the killing of *preborn human beings*, members of the human species developing in the womb. Worldwide, more than 10 million abortions are performed after 10 weeks’ gestation each year, including 200,000+ each year in the United States [11, 12].

*Homicide* refers to the morally unjust killing of another human person—that is, the violation of their serious moral right to life. This is homicide in the moral sense, not the legal sense, though the concepts overlap [10, p. 417]. Homicide is not equivalent to murder (a type of homicide) because murder requires the intent to kill another person and a high level of culpability, whereas homicide does not. For example, in ***Demolition*** when the demolition expert blows up the building she does not intend to kill another person—she substantially and unjustifiably risks doing so. If she in fact does kill another person, she is guilty of reckless homicide, not murder.

*Persons* refer to individuals with a high and equal moral status and the moral rights that go with it. For example, persons have a serious right to life, which implies that others have a stringent moral duty to not kill them. This is moral personhood rather than metaphysical personhood or legal personhood. These concepts must be distinguished to avoid confusion. A newborn baby, for example, is a person in the moral sense (of having equal moral status and rights) even if she is not yet a person in the descriptive metaphysical sense (of having self-awareness or psychological continuity).

*Harm* standardly refers to a ‘loss of welfare’ or ‘setback to interests.’ An act harms another person if it makes them worse off. It has proven difficult for philosophers to supply a more precise definition. Fortunately, we do not need one. We usually know harm when we see it.

*Foreseeable harm* (or expected harm) refers to probability-weighted harm—the product of a harm’s probability and magnitude.

To *criminalize* abortion is to make it a crime—to legally prohibit it and make those who commit it liable to conviction and a range of possible penalties if certain conditions are met, such as proof of guilt beyond a reasonable doubt.

Criminalization implies nothing about what the possible legal penalties should be (e.g. fines, medical license revocation, jail time) or whether someone liable to them should in fact be punished. But criminalization does imply liability. Who should be liable to punishment? If abortion is a crime, who should be the criminal?

The abortion provider should be liable to moderate penalties, but the woman should be presumptively exempt from prosecution. That is, antiabortion laws should target abortion provision rather than procurement or self-induced abortion. And they generally do [13] and have done so historically [14]. In the United States, for example, states that prohibit abortion explicitly exempt women or do so implicitly by targeting abortion providers. And they do so for justifiable reasons [2, pp. 108–111].

Consider three reasons. First, punishment should be proportionate to culpability. Many women seeking illegal abortions after 10 weeks’ gestation will likely be driven by desperate circumstances, intense fear, coercive pressure (explicit or subtle), or other mitigating circumstances. Many will also be uninformed or misinformed about the nature of abortion and preborn human beings (e.g. fetal development). Abortion providers, however, cannot invoke these mitigating factors and are generally more informed, hence their culpability is greater.

Second, it is plausible that punishing mothers or imposing harsh penalties on abortion providers would be counterproductive. For the criminal law to effectively prevent abortion, the prosecution needs proof of guilt to secure convictions. The woman’s testimony against the abortion provider is a key item of evidence. However, she will not be likely to testify if she herself may be prosecuted for her role. Moreover, the public is less willing to accept antiabortion laws that punish mothers or impose harsh penalties on providers. Such laws would likely create a political backlash and make juries less likely to convict offenders, which would undermine the perceived legitimacy and effectiveness of these laws.

Third, punishment should be proportionate to the seriousness of the crime. Abortion (if not verifiable homicide) is a form of endangerment, or so we argue in this paper. Endangerment is a lesser crime than homicide. If abortion is endangerment rather than homicide, then the penalties for abortion should be lesser penalties, comparable to the range of penalties for similar forms of endangerment. This would be another reason to impose moderate rather than harsh penalties. Later abortions would plausibly constitute more serious forms of endangerment, which would justify more serious penalties.

## Abortion substantially and wrongfully risks homicide

Now that *The Prevention Argument* has been laid out, I must support its premises.

Premise (1) can be supported by moral risk arguments, which claim that abortion is wrong because it risks homicide—the unjust killing of a person with rights. This moral risk could have various sources. It could be rooted in *moral uncertainty* (about the criteria of moral personhood or the ethics of killing), *empirical uncertainty* (about the capacities of preborn humans or the burdens of unwanted pregnancy), *metaphysical uncertainty* (about the criteria of personal identity over time, ensoulment, or the will of God), or all the above. Though such arguments have a long history [3], they are rarely developed in any detailed way [7, p. 11]. One exception is my *Do Not Risk Homicide Argument* [10], which argues that abortion is wrong after 10 weeks’ gestation (if not before then) because it substantially and unjustifiably risks homicide.

Since I am concerned here primarily with tracing the *legal implications* of this moral risk, I will motivate and expand upon my prior argument for it to motivate premise (1). Here is my *Do Not Risk Homicide Argument:*

(1.1) *Substantial Chance of Personhood*: There is a substantial chance that preborn human beings after 10 weeks gestation are persons with a serious right to life.
(1.2) If preborn human beings are in fact persons, then abortion is homicide, the unjust killing of a human person (exception: mother’s life cases).
(1.3) Therefore, abortion substantially risks committing homicide after 10 weeks gestation (exception: mother’s life cases). [From 1.1 and 1.2]
(1.4) *Do Not Risk Homicide*: It’s wrong to substantially risk committing homicide, unless there is overriding reason to take this risk.
(1.5) There is no overriding reason to substantially risk committing homicide through abortion after 10 weeks gestation (exception: mother’s life cases).
(1.6) Therefore, abortion is wrong after 10 weeks gestation (exception: mother’s life cases). [From 1.3, 1.4, and 1.5] [10, p. 416].

### A substantial chance of personhood

Premise (1.1) claims: “There is a substantial chance that preborn human beings after 10 weeks’ gestation are persons with a serious right to life.” *Personhood* refers to moral personhood (equal dignity and rights). A *substantial chance* of personhood refers to “*a more than 1 in 5 chance* (or probability higher than 0.2)” [10, p. 417]. In referring to chances and risk, I am talking about probability relative to the evidence available, not mere subjective confidence:

*Subjective probability* is all in your head: it is the level of confidence that you have in the truth of a proposition. For instance, some are supremely confident that preborn human beings are not persons. But we all know that our subjective confidence may not be warranted by the evidence available*. Evidential probability* in contrast is an objective notion that is sensitive to the evidence: it is the level of confidence in a proposition that would be warranted or justified by a body of evidence, such as the evidence available. For instance, given the evidence available, you should not be supremely confident that you will not kill anyone if you drive drunk. If you happen to be supremely confident and drive drunk, your subjective confidence would not get you off the moral hook. As the example indicates, evidential probabilities can be compelling and can have action-guiding moral implications, even if we cannot precisely quantify them [10, p. 417].

Given the evidence publicly available, there is a more than 1 in 5 chance that preborn human beings are persons after 10 weeks’ gestation (if not before then): “We make no attempt to precisely quantify this chance: such artificial precision eludes human beings who can seldom do more than roughly estimate the degree of support provided by the evidence” [10, p. 417].

Is there at least a 1 in 5 chance of personhood? The personhood literature suggests so. Jeff McMahan observes: “the main reason” why “abortion remains one of the most intractably controversial of all moral issues…is that the moral and metaphysical status of human embryos and fetuses is shrouded in darkness” [15, p. 3].

I have also presented six items of evidence, which together motivate a substantial chance of personhood [10, pp. 421–428]. Here, I will briefly review three items: widespread disagreement, the newborn-likeness of preborn human beings, and the common intuitive responses of women to their pregnancies and miscarriages. In considering the evidence, one must keep in mind the argument’s modest burden of proof. The evidence need not show that personhood is likely, only that it is not too improbable.

First, there is widespread disagreement in society and among the relevant experts about personhood after 10 weeks’ gestation. This disagreement falls roughly between a 40/60 and 60/40 split and is documented in the scholarly literature, abortion law, and public opinion polling. How should we respond to it? As I have argued, highly confident deniers of personhood should reduce their confidence. They need not give up their denial or suspend judgment, but they should reduce their confidence level—widespread disagreement shifts the probability of personhood. “After all, we are fallible, preborn personhood is a fairly difficult moral question—it is not like whether it is wrong to torture children for fun—and the other side’s answer is not clearly absurd. Perhaps we are missing something” [10, pp. 422–423].

Further, the epistemology of disagreement literature can be invoked for support: the dominant view is that widespread disagreement in society and among the relevant experts has evidential significance—highly confident parties to such a dispute should “conciliate” or lessen their confidence to some degree (other things being equal) [16]. For motivation, consider ***Demolition***. Suppose there was widespread disagreement among the demolition crew about whether a child was inside the building. Suppose they had similar evidence. How should they respond to their disagreement? Crew members highly confident that no child is inside the building should reduce their confidence and grant at least a non-negligible chance.

Second, consider the fact that preborn human beings older than 10 weeks’ gestation are much like premature newborns, who are persons. According to moral commonsense, newborns are persons with dignity and rights—they are treated as such in law and practice. For example, if someone enters a hospital and smothers a premature newborn who was born at 21 weeks’ gestation, he would be charged with criminal homicide, and rightly so. The fetal sciences and ultrasound imaging reveal that preborn humans older than 10 weeks’ gestation are much like premature newborns, both inwardly and outwardly, both physically and behaviorally. Since premature newborns are persons, the newborn-likeness of preborn humans older than 10 weeks is evidence that they are persons too. That is, unless there is some relevant difference between these two human populations that distinguishes (decisively) persons from nonpersons. However, as I have argued, the criteria of viability, birth, and sentience do not clearly do the distinguishing trick. And the personhood literature reinforces the point by consistently highlighting problems with these criteria.

Consider sentience and consciousness, for example. Some babies are born in a vegetative state but can gain sentience and the capacity for consciousness as a result of further development and therapy. If these capacities distinguish persons from nonpersons, such babies would not be persons with rights until after they receive therapy and gain the relevant capacities. This is hard to believe. Even if (hypothetically) sentience divided human persons from human nonpersons, it is uncertain when it begins—there is substantial empirical uncertainty and disagreement among fetal scientists. Moreover, there is a non-negligible chance that fetal sentience begins around 10–12 weeks’ gestation [17].^3^

Thus, I have argued that the newborn-likeness of preborn humans is evidence of their personhood—it boosts the probability. The evidence may not be decisive by itself, but it does suggest that their personhood isnot too improbable [10, pp. 425–427].

For motivation, consider ***Demolition***. Suppose the demolition expert is using infrared cameras to help keep the building site secure. Right before the deadline to blow it up, she looks at her monitor and sees infrared images of a small unidentified organism moving around the building. It is likely a stray animal, but it also physically and behaviorally resembles a small child. In this case, the child-likeness of the ambiguous figure constitutes evidence that a person is inside the building—it boosts the probability.

Third, consider the common intuitive responses of women to their pregnancies and miscarriages after 10 weeks’ gestation. Pregnant women ordinarily respond to and treat their preborn baby as a person rather than the moral equivalent of an animal, plant, or body part. For example, they refer to their ‘child’, ‘baby’, ‘son’, or ‘daughter’, name them, and love and care for them in thought, word, and deed, much like mothers care for their newborns. Moreover, women who experience a miscarriage after 10 weeks’ gestation typically grieve and mourn the loss of their irreplaceable child, much like we mourn the loss of newborns and other persons in our life [10, p. 428]. Even if the woman goes on to have another child, the miscarriage remains a tragic loss. If further evidence is desired, one should also consider the moral responses of women to the crime of fetal homicide—the killing of their preborn baby by an assailant.

What should be made of these common intuitive responses? I argue that they constitute evidence for the personhood of preborn humans. Why? Because common intuitive responses constitute (defeasible) evidence on any plausible epistemology of moral personhood. For example, the fact that one intuitively responds to newborns and cognitively disabled humans as persons is evidence of their personhood. So too the fact that pregnant women intuitively respond to their preborn babies as persons is evidence of their personhood. Moreover, these responses cannot easily be dismissed by critics as merely sentimental, confused, or otherwise unreliable. “Such responses are also fairly common among women with pro-choice moral and political commitments, which indicates that they are not simply mediated or biased by one’s prior commitments” [10, p. 428].

I have argued that the cumulative evidence (including the evidence reviewed here) establishes at least a 1 in 5 chance of personhood after 10 weeks’ gestation. I recognize the possibility that highly confident deniers of personhood could invoke some decisive argument to rebut the evidence and justify their continued confidence. Which arguments could do the trick for them? “After all, standard arguments (e.g., natural miscarriage arguments, twinning arguments, and embryo rescue cases) challenge the moral status of early human embryos, not preborn human beings older than 10 weeks gestation” [10, p. 423]. The main arguments available to them invoke mental capacity criteria of moral personhood such as self-awareness or rational agency to justify excluding preborn humans older than 10 weeks who do not yet possess these capacities [15, 18].

However, I cast doubt upon these mental capacity criteria of personhood (and one need only cast doubt, not refute). Such criteria conflict with moral commonsense by excluding human infants and a range of cognitively disabled humans from personhood and equal rights. Moreover, such criteria lack the explanatory power necessary to override moral commonsense, for example because they cannot easily explain why human persons have equal moral status when they possess the relevant mental capacities to different degrees. Postulating some threshold on the capacity scale—a “sufficient degree” of self-awareness or rational agency—“seems like an ad hoc device designed to preserve moral equality and…raises additional theoretical difficulties, such as where the cognitive threshold should be set and why there rather than elsewhere” [10, p. 425]. I conclude that confident denials of personhood after 10 weeks’ gestation cannot rationally rest on confidence in these mental capacity criteria [10, pp. 423–425]. The ground is too shaky.

### The good Samaritan argument

Premise (1.2) receives widespread support: “If preborn human beings are in fact persons, then abortion is homicide, the unjust killing of a human person (exception: mother’s life cases).” Personhood is central to abortion ethics and law [19]. However, there are dissenters. Judith Thomson [20] and a few others have argued that even if there is a 100% chance that preborn humans are persons with equal rights, abortion is still generally justified because it is analogous to refusing to be a good Samaritan—refusing to assist a needy person when the cost of doing so is high. It would be nice to donate one’s bodily assistance or one’s kidney to a needy person. But if the cost of doing so is high, it is surely permissible to refuse, even if otherwise the needy person will die. The needy person’s right to life does not entail a right to one’s bodily assistance. Thus, when one refuses bodily assistance to a needy person (e.g. the famous violinist) and he dies as a result, one does not violate his right to life. So too by analogy when a woman refuses bodily assistance to her child by having an abortion and the child dies as a result, she does not violate the child’s right to life. If the cost of assisting her child were small (hypothetically), it might be morally problematic for her to refuse, but it would not be unjust. Thus, abortion is generally permissible (given the high cost of unwanted pregnancy) and should be legal, even if preborn humans are persons. Courts and legislatures have not invoked the good Samaritan argument to justify abortion rights, but they could do so.

I presume—with most commentators—that the good Samaritan argument is flawed and will not work [10, pp. 418–419]. But I should here motivate the presumption.

The most compelling moral and legal objection is the *killing versus letting die objection* [15, 19]. Disconnecting from the violinist or refusing to donate an organ are standardly understood to be instances of letting die or refusing to assist, whereas abortion (at least typically) is an act of killing.

It is widely accepted that killing persons and letting them die (at least typically) are morally different acts and should be treated differently in law [15, 19]. In fact, all jurisdictions treat them differently. Though letting die and refusing to assist are frequently permissible, killing is generally wrong and illegal. That is, the moral and legal restrictions on killing are much more stringent. For example, it is permissible sometimes to let a patient die from a pre-existing illness by withdrawing medical treatment, but it does not follow that it is permissible to kill the patient by lethal injection. It is permissible to let a poor man die by refusing to donate a large sum of money to charity, but it does not follow that it is permissible to kill the poor man (e.g. to prevent him from taking the money). Thus, even if it is permissible to let the violinist die or to refuse to donate an organ, it does not follow that it is permissible and should be legal to kill preborn children in abortion. After all, morality and the law strictly prohibit killing innocent persons, except in cases like proportionate self-defense. Accordingly, if preborn humans are innocent persons with a right to life and abortion *kills them*, then it seems abortion should be strictly prohibited too, except in cases like proportionate self-defense [19].

Is abortion (at least typically) an act of killing? Consider the standard abortion methods used after 10 weeks’ gestation. The preborn human being is either dismembered with a powerful vacuum, dismembered limb by limb with grasping forceps, crushed by drug-induced contractions, or lethally injected with drugs that cause cardiac arrest. It is widely accepted that this is killing [15, p. 378].

Consider now a better analogy to abortion.^4^

***Separating conjoined twins***: Imagine you wake up in the morning and find yourself in bed next to your conjoined twin, who is unconscious. You were born as omphalopagus twins, joined at the abdomen. You were scheduled for a separation by skilled surgeons. But now there is a problem. Your twin has been diagnosed with a liver ailment which has rendered her unconscious (temporarily). The director of the hospital tells you, “Look, we’re sorry this happened. We know you are unhappy and wanted to be free from the burdens of being conjoined, and we were scheduled to surgically separate you, but to do so now would cause your twin to die from blood loss, because her liver ailment is preventing her blood from clotting. You would survive the separation, but she would certainly die. But don’t despair, it’s only for nine months. By then your twin will have recovered, and can be safely separated from you.” *Do you have to wait nine more months? Or would it be permissible to separate now?*

It would be morally wrong to separate now. It would also be illegal (criminal homicide), and rightly so [19, pp. 66–73]. The considerable burdens of remaining conjoined nine more months—the mental burdens, physical hardships, frustration of autonomy, etc.—would not justify killing your twin, morally or legally.

In some cases of conjoined twins, one twin is not viable on her own: she is completely dependent on her other twin’s body not just for nine months but for the rest of her life—separating them would kill her. Would it be permissible for the independent twin (without her sister’s consent) to separate and thereby kill her sister to avoid the lifelong burdens of remaining conjoined? It strongly seems not. It would also be illegal.

Unlike the violinist case and the organ donation case, **separating conjoined twins** is a better analogy to abortion because it involves *killing* an innocent person. The fact that a better analogy (or at least an equally good one) supports the opposite conclusion casts further doubt on the good Samaritan argument.

### The self-defense justification of abortion

Other dissenters to premise (1.2) invoke the self-defense justification: even if there is a 100% chance that preborn human beings are persons, abortion is still justified as killing in self-defense. Courts and legislatures have not invoked the well-established right to self-defense to justify abortion rights, but they could do so [21].

I presume—with most commentators—that this too will not work, at least when the mother’s life is not in danger [10 p. 319] [19]. But I should again motivate the presumption.

The most compelling objection to the self-defense justification is the *proportionality objection*: abortion (killing the preborn child) is generally not a proportionate response to the harm threatened. Both morality and law accept the proportionality requirement: the harm inflicted in self-defense must be proportionate to the harm thereby avoided. That is, the defensive harm must not be excessive in relation to the harm threatened, taking into account both the magnitude and probability of the harms. Most commentators think one should also take into account the moral nature of the source of the threatened harm: it is easier to justify killing a culpable aggressor than an innocent passive source of harm, like a small child [22]. When is killing, the most extreme defensive harm, a proportionate response? It is widely accepted that killing a culpable aggressor can be a proportionate response to avoid death or grave physical injury (e.g. paralysis).

Abortion is generally not a proportionate response to unwanted pregnancy, for three reasons. First, the maternal health risks of pregnancy are generally low-to-moderate, especially in countries with quality health care. Statistically there is roughly a 1 in 10,000 chance of maternal death [23]. Such a small risk never justifies defensive killing.

Second, the mental burdens, financial costs, social costs, and interference of unwanted pregnancy are just not the sorts of harms that can justify defensive killing. After all, born children pose comparable burdens and costs, but parents clearly cannot kill their children in self-defense (even if no one else will care for them).

Third, preborn children are innocent passive sources of potential harm. Paradigm cases of self-defense involve killing a culpable aggressor who is unjustly attacking the victim. If a culpable aggressor aims his gun at someone, intending to unjustly shoot them, then that person is justified in killing the aggressor in self-defense, provided there is no other way of avoiding the attack. However, unlike the culpable aggressor, the preborn child is not culpable nor an aggressor. Unlike the innocent aggressor (the brainwashed child soldier intending to unjustly shoot someone), the preborn child is not an aggressor at all—he is not doing anything, especially something unjust. He is just passively existing in the womb, the natural environment which we all inhabit early in life. Though he is a source of potential harm to the mother, he is neither culpable nor responsible for any harm his mere bodily presence might pose. Accordingly, it seems the harm he poses must be greater to justify killing him in self-defense (other things being equal).

For motivation, consider other innocent passive sources of harm:

***Child obstacle***: A driver is experiencing a heart attack and will probably die soon if she does not get to the hospital. She cannot get an ambulance to come and nobody else is available to take her. So she proceeds to rush herself to the hospital. On her way she encounters an injured child, who is blocking her only driving path. The child is alive and breathing but unresponsive. She cannot move the child or drive around him. Can she permissibly run over him (killing him), if necessary to save her life?

The child’s mere bodily presence poses a threat to the woman by blocking her only driving path to safety. Can she permissibly run over the child, if necessary to save her life? It is not obvious that she can. Suppose she is rushing to the hospital not to save her life but to avoid the low-to-moderate risks typical of pregnancy. Can she permissibly kill the child, or substantially risk doing so, if necessary to avoid these risks? It strongly seems not.

For these reasons, abortion (killing the preborn child) is generally not a proportionate response to unwanted pregnancy and thus cannot be justified as self-defense, at least when the mother’s life is not in danger.

### Do not risk homicide

Premise (1.4)—*Do Not Risk Homicide*—is a commonsense moral principle: “It’s wrong to substantially risk committing homicide, unless there is overriding reason to take this risk.” When the chance of committing homicide is substantial (greater than 1 in 5), one should not take the chance, unless one has strong overriding justification. As I have written elsewhere, “this is not to deny the moral relevance of lesser chances, nor is it to deny the fact that some chances are miniscule enough to be ignored….Rather, it is simply to observe that…a substantial chance of committing homicide is serious enough to require strong overriding reason to take it” [10, p. 419].

This paper’s opening cases motivate the principle. Notice also that the principle does not imply the wrongness of killing animals for food, mowing your lawn, etc., for such acts do not *substantially* risk committing homicide, even if they pose a minimal (nonzero) risk. The *reductio* objection is avoided [10, p. 418].

### No overriding reason to risk homicide

Premise (1.5) claims: “There is no overriding reason to substantially risk committing homicide through abortion after 10 weeks gestation (exception: mother’s life cases).” What overriding reasons could there be?

First, consider the *lesser risk justification*: abortion could be justified as the lesser moral risk. However, substantially risking homicide through abortion is clearly the greater moral risk. For the woman risks no serious wrongdoing by refraining from abortion and carrying her pregnancy to term, as Lockhart observes [4, p. 51]. It could be argued that by refusing to provide abortions after 10 weeks, providers risk interfering with reproductive liberty. However, unjustly killing an innocent person, even if less probable, is far worse than unjustly restricting another person’s reproductive liberty [10, p. 419]. The greater moral risk falls on the side of performing an abortion rather than refraining (for further motivation of this point, see the below section “The harm prevented outweighs the harm created”).

Second, consider the *staggering cost justification*: it could be permissible to substantially risk homicide to avoid a staggering cost to oneself. For example, can one substantially risk homicide to avoid their own death? Perhaps, if the risk is low though substantial and the probability of their own death is significantly higher [10, pp. 419–421].

Consider a case:

***Medical emergency***: A driver is experiencing a heart attack and will probably die soon if she does not get to the hospital. She cannot get an ambulance to come and nobody else is available to take her. So she proceeds to rush herself to the hospital. Anxious to save her life, she speeds and approaches a long red light at a blind intersection—her view of oncoming cars is completely obstructed. Should she run the red light, not knowing whether a car is coming the other way?

The staggering cost of probable death may justify the driver’s risky conduct. But if the cost to her is substantially smaller—if she faces not death but the prospect of financial insecurity, mental burdens, moderate physical hardships, etc.—then it would be wrong for her to endanger another person’s life. Our opening cases motivate the point.

Could the *staggering cost justification* justify abortion when the mother’s life is not in danger? No, because the costs of unwanted pregnancy, though considerable, are not sufficiently staggering (at least typically): they don’t approximate a cost like death the avoidance of which might justify substantially risking homicide (for motivation of this point, see the below section “The harm prevented outweighs the harm created”).

To sum up: abortion substantially and wrongfully risks committing homicide after 10 weeks’ gestation (exception: mother’s life cases). This is premise (1) of *The Prevention Argument*. Next, I turn to the normative legal implications of this moral risk.

## The harm principle

Premise (2) claims: “Homicide is a serious harm.” Though harm (like other important concepts) is difficult to define with precision, we usually know it when we see it. To smother a premature newborn baby, for example, is to seriously harm her [24]. (Ordinary parents know this). Homicide counts as a serious harm, if anything does. And the criminal law should prevent serious harms like homicide.

Premise (3), the *Harm Principle*, is a famous normative principle that guides criminalization decisions. According to it, lawmakers have good reason to criminalize conduct that wrongfully causes harm (or substantially and wrongfully risks causing harm) to other persons, if criminalization would prevent foreseeable harm and the harm prevented outweighs the harm created. This principle (or a very similar one) is widely accepted. Most agree that lawmakers should criminalize conduct that wrongfully harms or endangers other persons. Legal scholar Chris Clarkson explains:

It is widely accepted that the content of criminal law and the structure of its offences should be informed by the harm principle. Conduct should be criminalized if it causes harm to others. It is widely accepted that the definition of ‘harm’ for this purpose must include the risk of harm. Accordingly, most offences can be classified in one of two ways. First, there are harm-based or result crimes where the focus is on the resultant harm, for example, murder or manslaughter. Secondly, there are endangerment offences where the focus is on the risk-taking activity alone, irrespective of any resultant harm that may occur. Such conduct is criminalized because of its potential to cause harm and the seriousness of such offences is loosely linked to the likelihood (and potential gravity) of the harm materializing [25, p. 278].

Why should lawmakers criminalize endangerment? To prevent foreseeable wrongful harm. Antony Duff and Tatjana Hörnle explain:

If we have (as we must suppose we have) good reason to criminalize the reckless causation of harm to other people…we have just the same kind of reason to criminalize conduct that creates a significant and unjustifiable risk of such harm. …Those who are tempted or inclined to behave in such ways are as much in need of deterrence as those whose dangerous conduct actually causes harm, since they could well cause harm. Any plausible version of the Harm Principle…mandates the criminalization of conduct that threatens to cause harm as well as of conduct that actually causes harm [26, pp. 151–153].

The *Harm Principle* draws from Joel Feinberg’s canonical formulation of the principle: “It is always a good reason in support of penal legislation that it would probably be effective in preventing (eliminating, reducing) harm to persons other than the actor” [27, p. 26]. “Harm” in this formulation includes foreseeable or expected harm. Feinberg claims that “no responsible theorist denies the validity” of such a principle [27, p. 14].

Two clarifications are necessary. First, the *Harm Principle* claims that harm prevention is a “good reason” to criminalize, not a conclusive reason. There could be overriding reasons. The overall cost of criminalization, for example, could outweigh the harm preventive benefit. Second, harm prevention is “a good reason,” not the only reason. There might be other valid reasons to criminalize conduct, for example to prevent serious wrongs or the violation of human rights. Suppose a nurse euthanizes a competent patient suffering from pain at the end of his life, despite the patient’s expressed wish to live. This conduct should be criminalized (as it is), even if the killing of this patient (on some accounts) is not a serious harm. Why? To prevent the serious wrong of homicide, to protect the patient’s right to life. Accordingly, if abortion substantially risks committing homicide but not serious harm (given some contentious account of harm), lawmakers would still have a powerful reason to criminalize it, namely to prevent the foreseeable *wrong* of mass homicide. *The Prevention Argument* could then be suitably reframed in terms of wrong prevention rather than harm prevention.

Allow me to clarify the conditions of the *Harm Principle*.

First, *the wrongness condition*: “conduct that *wrongfully* causes harm (or substantially and *wrongfully* risks causing harm).” This normative requirement is widely accepted by legal theorists. Lawmakers should not criminalize conduct that *justifiably* causes or risks harm to other persons, such as just warfare or the speeding of emergency vehicles. Why not? For one reason, criminalizing conduct makes it punishable, but it is unjust to punish someone who has done nothing wrong.

Second, *the substantiality condition*: “conduct that…*substantially* and wrongfully risks causing harm.” Why must the risk be substantial? Consider ordinary driving and hunting, which risk serious harm to others. Should lawmakers criminalize such conduct (if proper precautions are taken)? No, because the risk in these cases is negligible, or too small to merit the expense and condemnation of the criminal law. But what counts as a *substantial* (or non-negligible) risk of harm?

The probability need not be high, as Feinberg observes [27, p. 190]. Conduct like drunk driving is rightly criminalized despite the low probability of harm eventuating. What such cases show is that the probability threshold varies with the magnitude of the potential harm: the greater the harm, the lower the probability threshold.

The probability also need not be precisely quantifiable. What is the probability that drunk driving will result in homicide? What about the conduct in our opening cases? Precise probabilities prove elusive in such cases, which indicates that the threshold is vague. Nevertheless, we can know when it has been crossed. Conduct that presents a more than 1 in 5 chance of committing homicide (like abortion plausibly does after 10 weeks’ gestation) clearly crosses the threshold—it poses a *substantial* risk of harm.

Third, *the prevention condition*: “if criminalization would prevent foreseeable harm.” If criminalization would not prevent the harm-causing or harm-risking conduct it targets, then it would be pointless from the standpoint of harm prevention. For example, if criminalization would not effectively prevent abortion—if it would just drive women to get illegal abortions at the same rate—then it would not be justified.

Finally, *the outweighing condition*: “if…the harm prevented outweighs the harm created.” Lawmakers should criminalize harm-causing or harm-risking conduct to prevent foreseeable harm. But what about the unintended harms criminalization might create? Zachary Hoskins observes: “it is difficult to make sense of a principle of criminalization grounded in a fundamental concern for harm reduction that did not also concern itself with the harms that are likely to stem from criminalization” [28, p. 631]. For example, suppose drug criminalization would fuel crime and divert law enforcement from more urgent priorities. If criminalization would create more foreseeable harm than it prevents, then it would not be justified. Any plausible harm principle must include an outweighing condition (at least implicitly).

## Criminalizing abortions would prevent them

Premise (4) claims: “Criminalizing abortions after 10 weeks gestation would effectively prevent them.” A common pro-choice objection insists otherwise: making abortions illegal will not stop them—women will continue to get abortions at the same rate. Criminalization would be pointless and thus is unjustified.

This objection fails on empirical grounds. The empirical evidence indicates that gestational limits prevent abortions from occurring after those limits. Of course, they do not prevent all abortions, just as homicide laws do not prevent all homicides, but they do prevent a rather large number. Direct evidence is provided by so-called ‘turnaway’ studies.

Consider the recent U.S. Turnaway Study conducted by pro-choice researchers. Over 1000 women seeking abortions at 30 clinics across the United States were periodically interviewed and followed for five years. Some received an abortion and others were ‘turned away’ or denied one because they were just over the gestational limit (in the second trimester) set by state law or abortion clinic policy. What happened to the women denied legal abortions? The large majority (70%) carried their pregnancy to term and kept their baby, even when abortion was legally available elsewhere [29, p. 16]. This is convincing evidence that gestational limits prevent abortions.

Similar studies have been done in other countries, with similar results. Diana Foster, director of both the U.S. Turnaway Study and the Global Turnaway Studies, summarizes the international evidence: “Scientists in other countries where abortion is legal—Bangladesh, Nepal, South Africa, and Tunisia—have found that among women who are denied legal abortion, about half get an abortion somewhere else and half carry the unwanted pregnancy to term” [30]. Foster admonishes her fellow abortion advocates: “stop saying that making abortion illegal won’t stop people from having them” [30].

## The harm prevented outweighs the harm created

Premise (5) follows from (1), (2), and (4). If abortions substantially risk homicide and criminalization would effectively prevent them, then criminalization would prevent foreseeable harm, namely mass homicide. How many potential homicides would criminalization prevent? Recall the preventive effectiveness of gestational limits. The U.S. Turnaway Study found that 70% of women carried their pregnancy to term when they were denied a legal abortion, even though abortion was legally available elsewhere. Recall that 200,000 + abortions are performed after 10 weeks’ gestation each year in the United States. If criminalization would prevent anywhere near 70% of these abortions, as the evidence indicates, then 100,000 + potential homicides would be prevented each year (by comparison, a total of 20,000 criminal homicides occur each year in the United States).

The foreseeable harm prevented is massive. If the practice of abortion is mass homicide (after 10 weeks gestation), then it is one of the worst mass homicides in human history. If two homicides are worse than one, the mass homicide of millions is catastrophic. It is also discriminatory in character. It is state-sanctioned mass killing that targets members of vulnerable groups, such as disabled persons.

Consider a comparable harm. Suppose there is a substantial chance (a more than 1 in 5 chance) that legalizing euthanasia would lead us down the slippery slope to mass homicide, on the order of 100,000 + unjust euthanasia killings each year in the United States, which target members of vulnerable groups like the elderly, psychiatric patients, and disabled persons. Suppose that criminalizing euthanasia would prevent this potential outcome. The foreseeable harm prevented in this case is massive and would give lawmakers a powerful reason to criminalize euthanasia. So too in the case of criminalizing abortion after 10 weeks’ gestation.

Premise (6) is the comparative harm assessment: “The foreseeable harm prevented by criminalization outweighs the harm created, especially if criminalization is combined with social policies that effectively support women.” Mass homicide is the main potential harm prevented, as has been shown. Would criminalization prevent other potential wrongful harms?

First, consider mass dehumanization—the denial of fundamental human rights protections to human persons. This is an awful harm and wrong committed by the state because legal recognition and protection is one of our most basic interests. As Barry and Tomlin suggest, “a state that failed to criminalize the killing of innocent, non-threatening people would seem to wrong its citizens quite seriously” [9, p. 460]. To appreciate the seriousness of mass dehumanization, we must keep in mind the historical record of societies treating ethnic minorities, indigenous peoples, disabled persons, and infants as nonpersons who can be permissibly killed to advance the interests of others. When the state permits the killing of preborn human beings in abortion, it denies them fundamental human rights protections, namely the right to life. Accordingly, legal abortion substantially risks mass dehumanization, since there is a substantial chance that preborn human beings are persons with human rights, at least after 10 weeks gestation. Criminalization would prevent this foreseeable harm and wrong committed by the state.

Second, consider the mass torture of innocent persons. Pain relief is not provided to preborn human beings during abortion procedures, for financial reasons and medical reasons (e.g. the added risk it poses to the mother). There is a consensus that preborn humans can feel pain by the third trimester (after 24 weeks). What about earlier? There is substantial empirical uncertainty and disagreement among fetal scientists, as a review of the literature confirms [31]. Stuart Derbyshire, one of the world’s leading specialists and a vocal supporter of abortion rights, recently reassessed the evidence and now maintains that preborn humans could feel pain as early as 12 weeks’ gestation. Accordingly, he argues commonsensically that abortion providers have moral reason to supply them with pain relief before abortion procedures [17]—that is, before they dismember, crush, or lethally inject them. I argue that this non-negligible chance of fetal pain, when conjoined with the substantial chance of moral personhood, implies that abortion procedures after 10 weeks’ gestation risk the *torture of innocent persons* with a serious right not to be tortured.^5^ “If researchers killed a newborn kitten or newborn baby by dismembering her or crushing her without pain relief, we would not hesitate to call it physical torture” [10, p. 429]. Even if the chance of torture is low around 10 weeks’ gestation, the chance increases every subsequent week and the potential harm is horrendous (torturing an innocent person). Criminalizing abortion after 10 weeks’ gestation would prevent this foreseeable harm.

What about the potential wrongful harms criminalization might create? I have argued that there is a substantial chance (a more than 1 in 5 chance) that abortion is homicide after 10 weeks’ gestation. But suppose there is also a substantial chance (given the evidence publicly available) that abortion is objectively permissible, indeed suppose there is a significantly higher chance. If so, *there is foreseeable wrongful harm on both sides of the criminalization decision*. The logic:

(Premise) There is a substantial chance that abortion is objectively permissible.

(Premise) If abortion is objectively permissible, then criminalizing it would unjustly impose hardships on women, interfere with their reproductive liberty, and unjustly punish abortion providers.

(Conclusion) Thus, there is a substantial chance that criminalizing abortion would unjustly impose hardships on women, interfere with their reproductive liberty, and unjustly punish abortion providers.

However, even if abortion has a higher chance of being objectively permissible than being homicide, the foreseeable harm prevented by criminalizing it would still outweigh the foreseeable harm created. Why? To understand, one must observe that the magnitude of a foreseeable harm depends on both the probability and magnitude of the harm. The probability matters: a 20% chance of death is more serious than a 1% chance. But the magnitude matters too: a 20% chance of death is more serious than an 80% chance of financial insecurity, mental burdens, moderate physical hardships, and moderate interference with one’s life. Why? Because death is catastrophic. The harm of death, even if less probable, vastly outweighs such moderate harms (in terms of its magnitude).

The foreseeable harm prevented by criminalizing abortion outweighs the harm created, for four reasons. First, the reproductive interference would be moderate. Second, the hardships imposed on women would generally be moderate. Third, the legal penalties imposed on abortion providers would be moderate. Fourth, the harm of mass homicide, even if less probable, vastly outweighs moderate reproductive interference, generally moderate hardships, and moderate legal penalties, especially if criminalization is combined with social policies that would effectively support women and thus mitigate the harm created. I now support these points in turn.

### The reproductive interference would be moderate

Reproductive liberty at its core is the freedom to determine whether and when to have children [32]. This liberty is not absolute. The vast majority of states restrict it by prohibiting abortions after a certain point in gestation, or for certain reasons. For example, 95% of countries restrict abortion in the second trimester. 83% of countries prohibit abortion in the second trimester if done for socioeconomic reasons [33]. Since reproductive liberty comes in degrees, restrictions of it do too. Prohibiting abortions after 10 weeks’ gestation would be a moderate restriction, resulting in a moderate loss of liberty. For women could still avoid unwanted pregnancy by using reliable contraception, natural family planning, or by acquiring a legal abortion *before* 10 weeks’ gestation (as most women do who get abortions). Women would still have extensive reproductive liberty.

### The hardships imposed on women would generally be moderate

The hardships imposed on women by criminalizing abortion (after 10 weeks’ gestation) would generally be moderate. Consider four empirically based reasons for thinking so.

First, women generally cite moderate hardships as their motivation for getting an abortion. The most common reasons cited are financial hardships, partner-related hardships, and interference with education and career opportunities [34–37]. For example, Finer et. al. [35] conducted the largest study in the United States and found that the most common reasons were: “Having a baby would dramatically change my life” (i.e. interfere with education, career, and ability to care for other children or dependents) (74%), “I can’t afford a baby now” (e.g. I’m unmarried, a student, can’t afford a baby and child care) (73%), and “I don’t want to be a single mother or am having relationship problems” (48%). It is worth observing that these hardships occur mostly after birth and are avoidable by placing the child in the care of others. In contrast, only a small minority of women cited hardships like concerns about their physical or mental health (12%), domestic abuse (2%) or rape (1%). More rarely did they cite them as their *most important reason*. For example, less than 4% cited maternal health concerns and less than 0.5% (0–4 out of 957) cited rape as their most important reason for seeking abortion. Moreover, the health problems women cited were typically moderate and treatable [35]. Statistics from other countries document similar behavioral patterns [34, 37].

Second, the hardships of unwanted pregnancy and parenthood are generally *avoidable*. Women and men can avoid them by avoiding risky sex (e.g. uncommitted sex), using reliable contraception, natural family planning, or practicing sexual self-control. If a woman for some reason cannot avoid unwanted pregnancy through these means, she could still avoid the main hardships after birth by placing the child in the care of others.

Third, the physical health risks of pregnancy are generally low-to-moderate. Though pregnancy entails physical burdens for the woman, maternal death and severe maternal harm are rare in countries with quality health care. Statistically, there is roughly a 1 in 10,000 chance of maternal death. For example, in the United States there are around 10 maternal deaths per 100,000 live births each year [23]. There is roughly a 1 in 100 chance of so-called “severe maternal morbidity” (SMM), which the U. S. Centers for Disease Control and Prevention (CDC) defines as “unexpected outcomes of labor and delivery that result in significant short- or long-term consequences to a woman’s health” [38, 39]. The most widely used statistical measure for SMM, developed by the CDC, is composed of 21 maternal health indicators (procedures and diagnoses) drawn from hospital records and codes at the time of childbirth. By far the most common indicator of SMM is that the mother received a blood transfusion of some amount, which is generally safe and does not reliably indicate true SMM because the blood loss in these cases is often non-life-threatening: “Some amount of blood loss during delivery is physiologically normal, and some experts argue that transfusion of a small amount of blood (e.g. 1 unit) may reflect routine preventive care” [38, p. 1891]. The CDC’s measure of SMM tries to reliably capture the presence of serious complications during childbirth. Though real, serious, and potentially life-threatening if not treated, such complications are treatable with quality health care (e.g. emergency obstetric care) and thus generally do not result in maternal death or severe maternal harm.

One must observe that abortion procedures come with their own health risks, and the risks are substantially and increasingly greater for second trimester and third trimester abortions [40]. When assessing the health risk imposed on women by criminalizing abortion, what matters is the *net risk*—the risk of pregnancy minus the risk of having an abortion. Even if carrying a pregnancy to term is risk*ier* than having an abortion after 10 weeks gestation, the net risk is generally low-to-moderate.

Consider data from the United Kingdom, for example. In 2022, there were 252,122 legal abortions, which had to be certified by two medical practitioners as lawful on specific statutory grounds. 98% (247,770 abortions) were performed for physical or mental health reasons (Ground C), with 99.9% of these performed for mental health reasons classified as F99 (“mental disorder, not otherwise specified”)—a catch-all category understood to include almost any reason [41]. In contrast, only 12 abortions were performed to “save the life of the pregnant woman” (Ground F) or “to prevent grave permanent injury to [her] physical or mental health” in an emergency situation (Ground G) [41]. 32 abortions were performed “to prevent grave permanent injury to [her] physical or mental health” in a non-emergency situation (Ground B) [41]. Finally, 77 abortions were performed because “continuance of the pregnancy would involve risk to the life of the pregnant woman greater than if the pregnancy were terminated” (Ground A) [41]. The legal ground for these latter abortions requires not that the pregnancy be life-endangering, only that it be riski*er* than having an abortion (which could be minimally risky). So, it is not clear how many of these 77 abortions were done for life-endangering reasons. Even if all they all were, however, the total number of abortions performed for life-endangering reasons would still be less than 100 abortions (less than 0.04% of all abortions) [41]. Other countries report similar data, such as the United States.

The data confirms that only a small percentage of women seeking abortion experience severe health risks, at least in countries with quality health care. Of course, these women do matter. In life-threatening cases, they could receive a legal abortion because virtually all countries allow abortion to save the mother’s life. But what if they face a serious risk to their *physical* *health* but not their *life*? If so, criminalizing abortion after 10 weeks’ gestation risks harming some of them, namely the small subset who (for some reason) would not be able to acquire a legal abortion *before* 10 weeks’ gestation. However, the numbers matter when assessing the magnitude of a harm. The unjust killing of 100,000 persons, for example, vastly outweighs the unjust burdening of 100 other persons with serious but non-life-threatening health risks. Accordingly, the mass homicide prevented by criminalization vastly outweighs the generally low-to-moderate health risks imposed on women by criminalization.

Fourth, the mental health risks of unwanted pregnancy are generally low, or non-existent. When assessing the mental health risk imposed on women by criminalizing abortion, what matters is the *net risk*—the risk of continuing an unwanted pregnancy minus the risk of having an abortion. The net risk is generally low, or non-existent. Why? Because a broad consensus exists that the mental health risks of unwanted pregnancy are no greater than the risks of having an abortion. The Royal College of Obstetricians and Gynaecologists, for example, reviewed the evidence and provides the following guidance: “Women with an unintended pregnancy should be informed that the evidence suggests that they are no more or less likely to suffer adverse psychological sequelae whether they have an abortion or continue with the pregnancy and have the baby” [42, pp. 45–4], [43]. Also consider the U.S. Turnaway Study, which followed women who were denied legal abortions. As noted, the large majority (70%) of women carried their unwanted pregnancy to term. One might expect them to suffer mental health harm as a result, such as anxiety or depression. However, they generally do not. Researchers found that women, though initially distressed, quickly came to terms with their unwanted pregnancy and did not experience substantial or lasting mental health harm: “carrying an unwanted pregnancy to term was not associated with mental health harm” [29, pp. 109, 128].

One final observation is worth making. When assessing the foreseeable harm to women of continuing an unwanted pregnancy, one should also take into account the *foreseeable benefit*. Many women choose to carry their initially unwanted pregnancy, give birth, and find themselves blessed with a loving relationship with their child, which they deem highly valuable if not priceless. As the U.S. Turnaway Study suggests, such beneficial outcomes are common for women [29, pp. 126, 204].

### The legal penalties imposed on abortion providers would be moderate

All criminal punishment is harmful and stigmatizes the offender. How harmful it is depends on the nature of the legal penalties imposed. If abortion is criminalized, it should be and probably would be criminalized with *moderate penalties* for abortion providers, as it generally is in states that criminalize it.^6^ Thus, the foreseeable harm to providers would be moderate. Moreover, the numbers matter. The number of providers punished if abortion is prohibited is likely to be small, especially compared to the number of potential homicides if abortion is permitted. For only a relatively small number of providers are actually prosecuted, convicted, and punished in states that prohibit abortion [13].

### Mass homicide outweighs the harm created

The harm of mass homicide is catastrophic and, even if less probable, vastly outweighs the moderate reproductive interference, generally moderate hardships, and moderate legal penalties created by criminalization. Consider three reasons for thinking so.

First, the harm of homicide vastly outweighs moderate hardships. Death does not merely burden someone, it completely deprives them of everything they have and ever will have. The magnitude of a harm is determined by factors like its duration (temporary or permanent?), extent (partial or total loss?), and reversibility (can it be mitigated or compensated for?). Death is permanent, total, and irreversible: you cannot compensate the victim because you cannot bring them back from the dead. In contrast, the hardships of unwanted pregnancy and parenthood consist of temporary, partial, and reversible losses of welfare. The main burdens of pregnancy itself (at 10 weeks’ gestation) will last only six more months, others can care for the child after birth, and the woman can still recover and live a worthwhile life, as most women do.

Second, the harm of homicide vastly outweighs moderate reproductive interference. For homicide deprives the victim of life and all liberty, not just a moderate degree of reproductive liberty. Homicide is worse than slavery too, which deprives the victim of all liberty, for slavery at least allows for life and the hope of a better future. Moreover, the loss of reproductive liberty would be mitigated or compensated for by social policies that promote reproductive liberty in other respects. For example, Friberg-Fernros points out that social policies that provide women with effective support would promote their freedom to determine whether and when to have children, for example by making pregnancy and parenthood less burdensome for them [7, pp. 56–57]. In contrast, the preborn child cannot be compensated for the loss of her life.

Finally, if one had to choose for oneself or one’s loved ones which harms to suffer, one would surely choose moderate hardships, moderate reproductive interference, and moderate legal penalties over death (i.e., being unjustly killed).

### Social support for women would mitigate the harm created

The foreseeable harm prevented outweighs the harm created especially if criminalization is accompanied by social policies that effectively support women and their children. Such policies include subsidized or free health care and childcare, job protection, parental leave, subsidized adoption, and safe haven laws that allow women to surrender their newborns to the care of others. The logic is clear: pro-woman, pro-childrearing social policies would mitigate the hardships imposed on women, enhance their reproductive liberty, and promote their social equality with men because, as feminist scholars have observed, “it is not chiefly pregnancy but more so *unavoidable childrearing* which threatens to socially disadvantage women [19, p. 99]. Such policies would thus mitigate the foreseeable harm caused to women by criminalizing abortion [7]. Accordingly, lawmakers have harm preventive reason to not only criminalize abortion but to do so while effectively supporting women and their children.

### Summing up the harm assessment

In this section, I argued for the comparative harm assessment in premise (6): the foreseeable harm prevented by criminalizing abortion, namely mass homicide, outweighs the harm created. Importantly, this is so even if abortions after 10 weeks’ gestation have a higher chance of being objectively permissible than being homicide. The additional foreseeable harms prevented by criminalizing abortion—namely, the mass dehumanization and torture of innocent persons—add further weight to the case. Next, I turn to an expected objection.

## The backstreet abortion objection

*The Backstreet Abortion Objection* runs as follows: even if criminalization would prevent many abortions after 10 weeks, it will not prevent them all. Some women in desperation will resort to dangerous illegal abortions, so-called ‘backstreet abortions.’ As a result, many women will die. If abortion after 10 weeks’ gestation turns out to be objectively permissible rather than homicide, these additional maternal deaths would be unjust harms caused by criminalization. When this foreseeable harm is added to the balance, it swings the other way—the foreseeable harm created outweighs the harm prevented.

*The Backstreet Abortion Objection* fails on empirical grounds, for two reasons. First, it is not clear that criminalization would increase the number of maternal deaths. If it would, we would expect countries that criminalize abortion to witness a subsequent increase. But they generally do not. For example, when Poland and Chile strictly prohibited abortion, their number of maternal deaths continued to drop [44]. One would also expect pro-life countries (which strictly prohibit abortion) to have higher rates of abortion-related maternal death than pro-choice countries (which liberally allow abortion). But there is no such documented pattern in the data.

Instead, the documented pattern is that high income pro-life countries have low rates of maternal death and low income pro-choice countries have relatively high rates. Abortion researcher Calum Miller observes that “mortality from induced abortion dropped to minimal levels before abortion was legalised in most Western countries…and is minimal to non-existent in high-income countries where abortion remains mostly illegal, such as Chile, Poland, Malta (pre-legalisation) South Korea, (pre-legalisation) Ireland and across Northern Africa and Western Asia” [44]. What explains data pattern? The best explanation is that the number of maternal deaths in a country is primarily determined by the quality of health care in that country (e.g. emergency care, post-abortion care)—abortion’s legal status makes little to no difference. “Once socioeconomic and infrastructural factors are taken into account, there appears to be little to no relationship between abortion legislation and abortion mortality” [44].

Second, even if criminalization would increase the number of maternal deaths, the additional number would predictably be extremely small and thus do little to counterbalance the massive harm prevented by criminalization.

To appreciate how small this number would be, consider *maternal mortality ratios*—that is, the rate of maternal death from miscarriage, abortion, or any pregnancy-related complication, per 100,000 live births. The World Health Organization (WHO) estimates that the European Union has a rate of 5 maternal deaths per 100,000 live births [45]. Most of these countries prohibit abortions after 12 weeks or before [46]. Maternal mortality ratios are also low in pro-life countries that both strictly prohibit abortion and have quality health care. For example, in Chile there are less than 15 maternal deaths and in Poland there are less than 2 maternal deaths per 100,000 live births [45]. Miller reviews the global evidence and observes that the rate of maternal death due specifically to either miscarriage or abortion (so-called “abortion mortality”^7^) is “now usually below 5 per cent of maternal deaths, and almost universally below 10 per cent,” which provides an upper limit on the subset of maternal deaths that could be due specifically to illegal abortion [47, p. 109]. What this means is that the rate of maternal death from either miscarriage or induced abortion (legal or illegal) is less than 1 death per 100,000 live births in places like the European Union, Chile, the United States, and other countries with quality health care. The rate of maternal death from specifically *illegal* abortion must be even smaller. This is so even in pro-life countries that strictly prohibit abortion.

Consider the European Union, 27 countries most of which prohibit abortion after 12 weeks or before [46]. In 2023, there were an estimated 3.67 million births in the European Union [48]. With a rate of 5 maternal deaths per 100,000 live births [45], this translates to an estimated 183 maternal deaths in 2023. The number of maternal deaths due specifically to either miscarriage or abortion (legal or illegal), as Miller observed, is less than 5% of this number—that is, less than 10 maternal deaths. Most of these deaths in turn were probably due to either miscarriage or legal abortion, so the number of maternal deaths due specifically to illegal abortion is extremely small, probably less than 5. By comparison, there are around one million abortions in the European Union each year.

Even if criminalizing abortion after 10 weeks’ gestation would increase the number of maternal deaths, the additional number would predictably be extremely small and thus do little to counterbalance the massive foreseeable harm prevented by criminalization. Of course, the wrongful death of just one woman would be tragic. But the unjust killing of a massive number of children would be catastrophic. If there is foreseeable wrongful harm on both sides, it is obvious where the balance tilts.

## Does the cost of criminalization outweigh the benefit?

The final hurdle for criminalization decisions is an overall cost–benefit assessment, where ‘costs’ and ‘benefits’ are understood broadly. Does the cost of criminalization outweigh the benefit? That is, do the reasons against criminalization override the reasons for it? Consider a stock example. Should one criminalize adultery to prevent the harm it causes? Its enforcement would predictably result in a substantial loss of privacy and divert resources from more urgent priorities. If the cost outweighs the harm preventive benefit, then criminalizing adultery would not be justified.

The costs of criminalization can include the material, social, and moral costs of enforcement, investigation, and punishment. The benefits can be preventive: criminalization could prevent foreseeable wrongful harms like mass homicide through mechanisms of deterrence, incapacitation, and social norm shaping. The benefits can also be retributive: there could be independent moral value in giving culpable abortion providers the punishment they deserve for substantially and unjustifiably risking homicide. Finally, the benefits can be expressive: the criminal law communicates, and its messages can be valuable. When the state criminalizes drunk driving, abortion after 10 weeks’ gestation, or other forms of endangerment, it communicates the moral gravity of such acts and reinforces social norms prohibiting them. Of course, there is theoretical debate about which benefits have priority. Consequentialists tend to argue that harm prevention is the only justification for criminalization. But most legal theorists are pluralists, allowing for preventive benefits, retributive benefits, and expressive benefits. If retributive benefits and expressive benefits are allowed, the case for criminalizing abortion would be stronger.

The main benefit of criminalizing abortion after 10 weeks' gestation is that it would prevent the foreseeable wrongful harms of mass homicide, mass dehumanization, and the mass torture of innocent persons. The foreseeable wrongful harms created by criminalization—moderate reproductive interference, the imposition of generally moderate hardships on women, the imposition of moderate legal penalties on abortion providers—fail to outweigh this benefit. This is so especially if criminalization is combined with social policies that would effectively support women and thus mitigate their hardships, enhance their reproductive liberty, and promote their social equality with men. Could additional costs tip the balance the other way? It is not clear what those costs could be. *The* *Prevention Argument* challenges supporters of legal abortion to identify costs of criminalization that outweigh the enormous harm preventive benefit.

## Conclusion

If abortion substantially committing risks homicide after 10 weeks’ gestation (as it plausibly does), then lawmakers should criminalize such abortions to prevent the foreseeable harm of mass homicide. *The Prevention Argument* is a powerful secular argument and deserves a hearing, for two reasons.

First, the argument relies on widely accepted normative principles, such as the harm principle. Everyone agrees that we should criminalize conduct that wrongfully endangers innocent persons, for example conduct that substantially and wrongfully risks committing homicide. Second, the argument has a modest burden of proof. It is not necessary to establish whether abortion *is* homicide, only whether it *substantially risks* homicide. If abortion presents this risk of harm, then lawmakers have a powerful reason to criminalize it, which is not easily outweighed by countervailing costs.

Could the argument be extended to earlier abortions, before 10 weeks’ gestation? It could if such abortions also substantially risk committing homicide, if criminalization would effectively prevent them, if the foreseeable harm prevented outweighs the harm created, and the harm preventive benefit outweighs the overall cost of criminalization. Such questions deserve serious consideration, given how much is at stake.

## Footnotes

1 This uncertainty could be due to moral uncertainty (about the child’s personhood and rights or the ethics of killing), empirical uncertainty (about the child's prognosis or the burdens of caring for the child), or both.

2 The U. S. Supreme Court, for example, ruled in *Roe v. Wade* (1973) that preborn human beings are not persons in the legal constitutional sense—acknowledging that if they were persons with a constitutionally protected right to life, then abortion would violate it. Interestingly, the court professed substantial uncertainty about their moral personhood: “We need not resolve the difficult question of when life begins. When those trained in the respective disciplines of medicine, philosophy, and theology are unable to arrive at any consensus, the judiciary, at this point in the development of man’s knowledge, is not in a position to speculate.” However, the court denied preborn humans basic legal protection by recognizing a constitutional right to kill them in abortion and thus implicitly denied their moral personhood and rights (at least before the point of “viability”). This implicit denial of personhood in *Roe v. Wade* and later abortion decisions is widely observed by legal commentators [1], [2, pp. 26-31].

3 Stuart Derbyshire, one of the world’s leading specialists, recently reassessed the evidence and now maintains that fetal sentience could emerge as early as 12 weeks gestation [17]. Since estimations of gestational age (“weeks gestation”) are routinely off by ± 2 weeks in the real world of medical practice, I infer that there is a non-negligible chance of fetal sentience around 10 weeks gestation [10, p. 427].

4 This case is patterned on Thomson’s violinist case [20].

5 Since estimations of gestational age (“weeks’ gestation”) are routinely off by ± 2 weeks in medical practice, I infer that the risk of torturing innocent persons extends from 12 weeks’ gestation down to 10 weeks’ gestation [10, pp. 428–429].

6 See the above section “The prevention argument” for reasons why antiabortion laws should (and generally do) target abortion providers rather than the women who procure abortions from them.

7 It is hard for medical examiners to distinguish between maternal deaths due to miscarriage (“spontaneous abortion”) and those due to induced abortion (legal or illegal), so these deaths get lumped together statistically under the umbrella of “abortion mortality.” Abortion mortality rates provider an upper limit on the rate of maternal death that could be due specifically to induced illegal abortion.
